# Surgical trauma is associated with renal immune cell activation in rats: A microarray study

**DOI:** 10.14814/phy2.15142

**Published:** 2021-12-09

**Authors:** Michael Hultström, Di Peng, Mediha Becirovic Agic, Claire G. Cupples, William A. Cupples, Nicholas Mitrou

**Affiliations:** ^1^ Department of Medical Cell Biology Integrative Physiology Uppsala University Uppsala Sweden; ^2^ Department of Surgical Sciences Anesthesia and Intensive Care Medicine Uppsala University Uppsala Sweden; ^3^ Department of Molecular Biology and Biochemistry Simon Fraser University Burnaby British Columbia Canada; ^4^ Department of Biomedical Physiology and Kinesiology Simon Fraser University Burnaby British Columbia Canada; ^5^ Department of Surgery University of Western Ontario London Ontario Canada

## Abstract

Acute kidney injury (AKI) is a common perioperative complication that is associated with increased mortality. This study investigates the renal gene expression in male Long–Evans rats after prolonged anesthesia and surgery to detect molecular mechanisms that could predispose the kidneys to injury upon further insults. Healthy and streptozotocin diabetic rats that underwent autoregulatory investigation in an earlier study were compared to rats that were sacrificed quickly for mRNA quantification in the same study. Prolonged surgery caused massive changes in renal mRNA expression by microarray analysis, which was validated by quantitative real‐time PCR with good correlation. Furthermore, bioinformatics analysis using gene ontology and pathway analysis identified biological processes involved in immune system activation, such as immune system processes (*p* = 1.3 × 10^−80^), immune response (*p* = 1.3 × 10^−60^), and regulation of cytokine production (*p* = 1.7 × 10^−52^). PCR analysis of specific cell type markers indicated that the gene activation in kidneys was most probably macrophages, while granulocytes and T cell appeared less activated. Immunohistochemistry was used to quantify immune cell infiltration and showed no difference between groups indicating that the genetic activation depends on the activation of resident cells, or infiltration of a relatively small number of highly activated cells. In follow‐up experiments, surgery was performed on healthy rats under standard and sterile condition showing similar expression of immune cell markers, which suggests that the inflammation was indeed caused by the surgical trauma rather than by bacterial infection. In conclusion, surgical trauma is associated with rapid activation of immune cells, most likely macrophages in rat kidneys.

## Graphical abstract

Network of genes activated by surgery that may predispose the kidney to further damage.

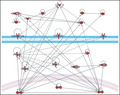

## 1 INTRODUCTION

Recent studies have shown that surgery, irrespective of type, induces a systemic immune response that may predispose other organs to injury and at a later stage also failure (Fidalgo et al., 2011; Terrando et al., 2010). Surgery triggers inflammatory cascades throughout the body, including pro‐inflammatory cytokine secretion, immune cell recruitment, and increased vascular permeability (Wan et al., 2007). One of the most common complications in patients exposed to surgery is acute kidney injury (AKI), a complication associated with increased mortality, poor outcome, and prolonged recovery time (Chertow et al., 2005; Mao et al., 2013; Rewa & Bagshaw, 2014). The major focus of previous studies has been on AKI after cardiac surgery. According to RIFLE criteria (an acronym comprising Risk, Injury, and Failure; and Loss, and End‐stage kidney disease), 19.3% of patients who had undergone cardiac surgery suffered from renal impairment after cardiac surgery and patients classified as RIFLE‐Failure had a 90‐day mortality rate of 32.5% (Dasta et al., 2008; Lagny et al., 2015). More importantly, increasing evidence has revealed that surgical trauma can lead to AKI even without direct intervention on the kidney. In a study consisting of 10615 patients undergoing orthopedic surgery, AKI affected up to 11% of patients, including increased long‐term mortality (Bell et al., 2015). This indicates that some surgery‐induced change in renal function may predispose to AKI.

Although AKI is a common form of postoperative organ failure, the direct cause and underlying mechanisms remain unclear. Pathogenesis of AKI has mainly been investigated in ischemic and septic animal models. Hemodynamic injuries including tubular obstruction, vascular dysfunction, and necrosis are commonly found in animals with ischemic AKI (Bonventre & Yang, 2011). In AKI induced by sepsis, local immune system is highly activated, triggering pro‐inflammatory cytokine secretion and leukocyte infiltration, ultimately leading to apoptosis and endothelial damage (Doi et al., 2009). Indeed, immune cell activation is a major mechanism behind the development of AKI (Jang & Rabb, 2015). To date, there is no direct intervention for AKI after surgery, and a better understanding of underlying molecular and cellular mechanism is required in search for therapeutic targets.

Diabetes mellitus (DM) is one risk factor of developing AKI, both alteration in renal blood flow and glomerular hypertension are found in diabetic animals (Rosolowsky et al., 2008; Zatz et al., 1986). In our previous study, we showed that renal autoregulation was transiently impaired in the early stage of DM induced by streptozotocin (STZ) (Mitrou et al., 2015). Interestingly, it also suggested a surgery‐induced inflammatory response in the kidney that was independent of DM, which is further explored in this study. To evaluate change in renal gene expression after surgery, we performed microarray analysis and qPCR on kidney tissue from rats after 3 hours of surgery with anesthesia and open abdomen that were compared with quickly sacrificed control animals. Diabetic animals from the original study (Mitrou et al., 2015) were included to determine if diabetes exacerbates the surgery‐induced gene expression changes.

## 2 MATERIALS AND METHODS

### 2.1 Animals

All in vivo experiments were conducted in Department of Biomedical Physiology and Kinesiology, Simon Fraser University, Canada. All experiments were approved by the University's Animal Care Committee (IACUC). Male Long–Evans rats (aged 12–16 week) were purchased from Harlan Laboratories (Livermore, CA), housed in groups of 2–6 in a 12:12‐h light–dark cycle, and provided with ad libitum access to standard rat chow (LabDiet 5001) and distilled water as described previously (Mitrou et al., 2015). Diabetes mellitus was induced with 60 mg/kg of streptozotocin injection into the tail vein (STZ). Subcutaneous insulin implants (Linplant, LinShin Canada, Scarborough, ON, Canada) were inserted (1/2 implant) at the time of STZ injection to keep blood glucose (BG) at about 20 mM and to allow the rats to maintain their body weight (Lau et al., 2009; Sima et al., 2012). Rats had free access to 5% sucrose in water for the first 24 h after STZ to guard against hypoglycemia in case STZ was ineffective. Body weight (BW) and BG were measured three times per week in each rat beginning the day before STZ injection until the day of the terminal experiment. Originally, surgery with prolonged anesthesia (isoflurane 4% during surgery followed by ~2%, 3‐4 h) was performed on both healthy (*n* = 7) and diabetic rats (*n* = 7). Surgery was performed 4 weeks following diabetes induction and in age‐matched controls. It included tracheal cannulation and mechanical ventilation, femoral venous and arterial cannulation, left kidney exposure through a subcostal incision, and placement of a renal arterial flow probe (TS420; Transonic Systems). Rats allocated to the no‐surgery group were sacrificed immediately following nephrectomy (*n* = 6 STZ and *n* = 7 healthy). All the sample preparations and procedures were performed as described in the original study (Mitrou et al., 2015).

To exclude the effect of infection, separate experiments were performed in non‐diabetic rats, comparing standard clean laboratory surgery with surgery performed under fully sterile conditions. RNA‐later stabilized kidney tissue from sterile surgery (*n* = 4) and non‐sterile surgery (*n* = 4) was collected and frozen until used.

### 2.2 Microarray expression analysis

RNA was quantified and checked for purity using a NanoDrop (Thermo Fisher Scientific) spectrophotometer, and quality was evaluated using the Agilent 2100 Bioanalyzer system (Agilent Technologies Inc). Two hundred and fifty nanograms of total RNA from each sample was used to generate amplified and biotinylated sense‐strand cDNA from the entire expressed genome according to the GeneChip® WT PLUS Reagent Kit User Manual (P/N 703174 Rev. 1, Affymetrix Inc.). GeneChip® ST Arrays (GeneChip® Rat Gene 2.1 ST 24‐Array Plate) were hybridized for 16 h in a 45°C incubator, washed and stained and finally scanned with the GeneTitan® Multi‐Channel (MC) Instrument, according to the GeneTitan Instrument User Guide for Expression Arrays Plates (PN 702933 Rev. 2, Affymetrix Inc.). The dataset has Array Express Accession number: E‐MTAB‐3661 (http://www.ebi.ac.uk/arrayexpress/).

### 2.3 Quantitative real‐time PCR

Both original and sterile/non‐sterile groups were used for qPCR validation of the gene expression. Nine inflammatory cell and endothelial marker genes were selected for qPCR validation: *Ccr4* (C‐C motif chemokine receptor 4); *Cxcr3* (C‐X‐C motif chemokine receptor 3); *Gja1* (gap junction protein, alpha 1, Connexin 43); *Gja4* (gap junction protein, alpha 4, Connexin 37); *Itfg1* (integrin alpha FG‐GAP repeat containing 1); *Ly6g6c* (lymphocyte antigen 6 family member G6C); *Nos2* (nitric oxide synthase 2); *Nos3* (nitric oxide synthase 3); and *Ptgs2* (prostaglandin‐endoperoxide synthase 2). Total mRNA was extracted from 30 mg kidney tissue of each individual with RNeasy® Mini kit (Cat No. 74104, QIAGEN), and purity and concentration were assessed using Nanodrop 2000 (Thermo Fisher). TaqMan probes (TaqMan® Gene Expression Assays, Thermo Fisher) and SYBR kit (QuantiNova SYBR Green PCR Kit, QIANGEN) were used for qPCR. The amplification was performed on QuantStudio 5 (Thermo Fisher). The target gene expression was normalized by 18S ribosomal RNA. Data are presented as Ct–Ct value.

### 2.4 Immunohistochemistry

Immunohistochemistry was used to quantify resident immune cells in the kidneys. The leukocyte common antigen, CD45 (Abcam, UK, ab10558, 1:100 in 2.5% goat serum), was used to estimate the total number of leukocyte lineage cells in the kidneys. The T‐cell co‐receptor CD3 (Abcam, UK, ab5690, 1:100 in 2.5% goat serum) was used to quantify T cells, and the macrophage marker CD68 (Abcam, UK, ab125212, 1:500 in 2.5% goat serum) was used to quantify macrophages. Formalin‐fixed and paraffin‐embedded kidney sections (5 µm) were deparaffinized in xylene (VWR, Sweden) and rehydrated in decreasing concentrations of ethanol (100, 95, 90, 80, and 70%). For CD45 and CD3 antigen retrieval, sections were boiled in citric acid pH 6.0, 3 × 4 min in a microwave oven at 900 W. For CD68 antigen retrieval, sections were boiled in DIVA Decloaker (Biocare Medical) using 2100 Antigen Retriever (1 cycle, Aptum Biologics, UK). After cooling, the sections were washed in PBS and blocked in 5% goat serum (Vector Laboratories, Sweden) for 30 min at room temperature, and incubated in primary antibody at 4ºC over night. Following incubation, the sections were washed in PBS and endogenous peroxidase was blocked using 0.35% hydrogen peroxide solution (Sigma, Sweden, 15 min at room temperature). The sections were washed once again in PBS and incubated in HRP‐conjugated secondary antibody (Abcam, UK, ab6721, 1:400 in PBS) for 1 hour at room temperature. Following a wash in PBS, the sections were incubated in DAB substrate containing 50 mM Tris, pH 7.6, 2% 3,3‐diaminobenzidine (Sigma, Sweden), and 0.3% hydrogen peroxide (100:2.5:1). The reaction was terminated by rinsing the sections in running tap water for 5 min. Mayer's hemalum solution was used for counterstaining, and the sections were dehydrated in increasing concentrations of ethanol (70, 80, 90, 95, and 100%). The number of positively stained cells was counted on 10 randomly chosen fields at 20× for each section using ImageJ (Mao et al., 2013).

### 2.5 Statistic and bioinformatic analysis

Data were normalized using the robust multi‐array average (RMA, Expression Console, Affymetrix) method first suggested by Li and Wong, 2001 (Irizarry et al., 2003). Subsequent analysis of the gene expression data was carried out using R and Bioconductor (Gentleman et al., 2004; R Development Core Team, 2014). Differentially expressed genes were tested using empirical Bayes moderated *t* test (eBayes) from the limma package (Gentleman et al., 2006; Smyth, 2004), and corrected for multiple testing using false discovery rate (FDR) (Benjamini, 1995). High‐level analysis was performed using Ingenuity Pathway Analysis (IPA, QIAGEN Redwood City, www.qiagen.com/ingenuity) and gene ontology enrichment analysis software toolkit (GOEAST) (Zheng & Wang, 2008). The dataset was compared with in‐common differentially expressed genes from a previous comparison of six models of AKI (Hultstrom et al., 2018). Significant overlap was tested using the exact binomial test (Clopper & Pearson, 1934). The Student's *t* test was performed for comparisons between surgery/no‐surgery and sterile/nonsterile, respectively, as these experiments were performed separately. Data are shown as boxplots with individual data points as overlay and tabled as (mean ± SEM). *p* < 0.05 was considered statistically significant. Statistical calculations were performed in R version 3.3.1 (R Development Core Team, 2014).

## 3 RESULTS

### 3.1 Samples and RNA quality

For the microarray analysis, 12 samples from rats that were sacrificed quickly (no‐surg) and 12 samples from rats that underwent longer anesthesia and surgery (surg) were used. The surgery group involved a 3–4 h experiment involving major surgery followed by assessment of renal autoregulation (surg) before sacrifice and tissue collection as described in detail in the original paper (Mitrou et al., 2015). Half of each group were diabetic for 4 weeks before sacrifice (STZ). The average total RNA concentration was 1336 ± 71 µg/nl with good purity and RNA quality with a RIN average of 7.69 ± 0.12. In the samples included in the microarray experiment there was a slight difference in RIN (no‐surg: 7.44 ± 0.17 and surg: 8.15 ± 0.1). There were no differences in RIN between STZ and no‐STZ groups within the same surgical group.

### 3.2 Differential expression

**FIGURE 1:**
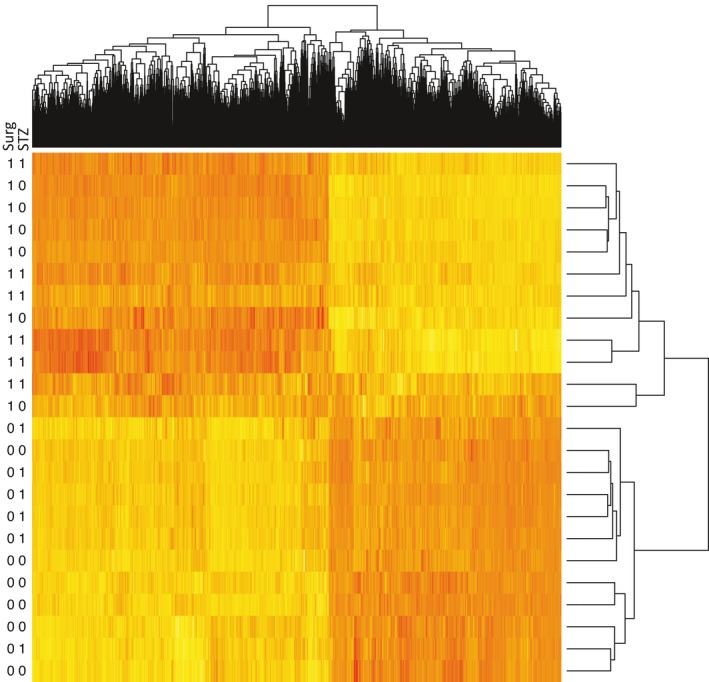
Heatmap and hierarchical clustering of differentially expressed genes and samples showing the very substantial difference between animals that underwent prolonged surgery (surg 1, *n* = 12), compared to animals that are euthanized quickly (surg 0, *N* = 12). This difference completely over‐shadowed any effect of streptozotocin‐induced diabetes mellitus (STZ 1 vs. no‐STZ 0)

There was no significant differential gene expression caused by 4 weeks of STZ‐induced diabetes, while many genes were changed by surgery whether rats were diabetic or not. There were 2673 differentially expressed genes between no‐surg and surg groups that were seen both with and without STZ. When filtering by fold change larger than 2 (i.e., absolute Log2‐ratio >1) there were 1127 (*p* = 5.6 × 10^−13^, i.e., the probability of randomly picking 5759, and 7074 genes out of 29489 and there being 1127 genes in‐common between the two picks) and 1002 in‐common genes, respectively (*p* < 2.2 × 10^−16^). Next, the 5759 differentially expressed genes were plotted as a hierarchically clustered heatmap across all samples. This clearly showed that all samples segregated according to surgery, but that STZ did not cause any visible patterns (Figure 1).

### 3.3 Pathway analysis

In order to investigate the function of the identified genes, the dataset was imported into Ingenuity Pathway Analysis (IPA) providing pathway information on 19467 genes in total and, 989 of the in‐common genes. Pathway analyses were performed on both datasets with similar top hits indicating inflammatory responses. Five hundred and eighty‐eight of these genes could be connected into a single network that had several prominent regulatory hubs such as the acute phase response regulators JUN, FOS, MYC, NFKB, IRF, STAT1, STAT3, PTGS2, NOS2, and many others.

### 3.4 Gene ontology enrichment analysis

The 989 in‐common genes from the comparison of surg and no‐surg with or without diabetes were imported to GOEAST as gene symbols, which produced 1446 enriched gene ontology terms across the three main ontologies, biological process (Table 1), cellular compartment (Table 2), and molecular function (Table 3). Using a *p* < 0.001 limit for enrichment, biological process had 727, cellular compartment had 28, and molecular function had 132 significant terms, respectively.

**TABLE 1:** Gene ontology—biological process

GOID	Term	Level	*p*
GO:0002376	Immune system process	1	1.31E−80
GO:0050896	Response to stimulus	1	3.82E−65
GO:0006955	Immune response	2	1.21E−60
GO:0009607	Response to biotic stimulus	2	1.49E−58
GO:0006950	Response to stress	2	3.91E−46
GO:0051707	Response to other organism	3	8.36E−61
GO:0006952	Defense response	3	4.86E−53
GO:0009611	Response to wounding	3	5.46E−51
GO:0001817	Regulation of cytokine production	4	1.65E−52
GO:0034097	Response to cytokine stimulus	4	8.51E−46

Note

Top hits in the gene ontology for biological process out of 727 enriched terms (*p* < 0.01) based on the differential expression from microarray experiments comparing prolonged surgery (*n* = 12) to quick euthanasia (*n* = 12) and analyzed using empirical Bayes and corrected using the false discovery rate.

**TABLE 2:** Gene ontology—cellular component

GOID	Term	Level	*p*
GO:0005576	Extracellular region	1	5.45E−22
GO:0016020	Membrane	1	1.21E−10
GO:0044421	Extracellular region part	2	4.88E−24
GO:0005615	Extracellular space	3	3.11E−32
GO:0071944	Cell periphery	3	1.37E−19
GO:0009986	Cell surface	3	1.08E−16
GO:0005886	Plasma membrane	4	2.44E−16
GO:0044459	Plasma membrane part	5	2.56E−12
GO:0009897	External side of plasma membrane	6	1.50E−12
GO:0042612	MHC class I protein complex	7	2.73E−12

Note

Top hits in the gene ontology for Cellular Component out of 28 enriched terms (*p* < 0.01) based on the differential expression from microarray experiments comparing prolonged surgery (*n* = 12) to quick euthanasia (*n* = 12) and analyzed using empirical Bayes and corrected using the false discovery rate.

**TABLE 3:** Gene ontology—molecular function

GOID	Term	Level	*p*
GO:0004872	Receptor activity	1	1.19E−15
GO:0005515	Protein binding	2	1.96E−14
GO:0003700	Sequence‐specific DNA‐binding transcription factor activity	2	2.56E−13
GO:0005102	Receptor binding	3	3.23E−19
GO:0005125	Cytokine activity	4	4.75E−27
GO:0005126	Cytokine receptor binding	4	7.98E−25
GO:0004896	Cytokine receptor activity	5	9.00E−21
GO:0042379	Chemokine receptor binding	5	2.85E−16
GO:0004830	Tryptophan‐tRNA ligase activity	6	8.97E−18
GO:0008009	Chemokine activity	6	4.18E−16

Note

Top hits from the gene ontology molecular function out of 132 significantly enriched terms (*p* < 0.01) based on the differential expression from microarray experiments comparing prolonged surgery (*n* = 12) to quick euthanasia (*n* = 12) and analyzed using empirical Bayes and corrected using the false discovery rate.

### 3.5 Comparison with known AKI genes

**FIGURE 2:**
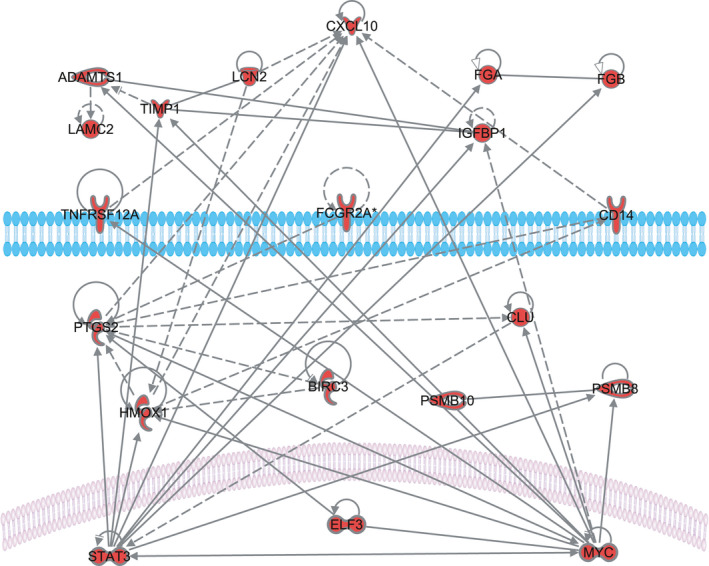
Connected network of genes that are both part of the 588‐gene network in the present investigation and are previously found to be in‐common for experimental models of AKI. This indicates that surgery without direct renal insult activates similar genetic programs as those seen in other forms of AKI

In order to focus on known AKI genes, the data were compared with in‐common genes for AKI models identified previously (Hultstrom et al., 2018). Out of the connected network of 114 genes in that study, 113 were assayed in the presented dataset. Out of the in‐common 989 differentially expressed genes that were found in both STZ and no‐STZ animals, 68 were found in the published AKI network showing that gene sets share common central features (*p* < 2.2E‐16). These 68 include well‐known AKI markers such as lipocalin‐2 (LCN2 or NGAL) indicating renal stress or incipient AKI. The in‐common genes also include the most well‐connected genes in both datasets MYC, STAT3 of the nuclear genes, cyclooxygenase 2 (PTGS2) and heme oxygenase 1 (HMOX1) in the cytosol and the interferon inducible, chemokine ligand 10 (CXCL10) in the extracellular region. This may indicate that part of the gene activation seen in AKI, and presumably connected to tissue damage, is already activated by surgical trauma, without direct renal injury. Of the 68 in‐common genes, 19 could be connected in a regulatory network that may be important for AKI priming in surgical trauma (Figure 2).

### 3.6 Quantitative real‐time PCR validation

**FIGURE 3:**
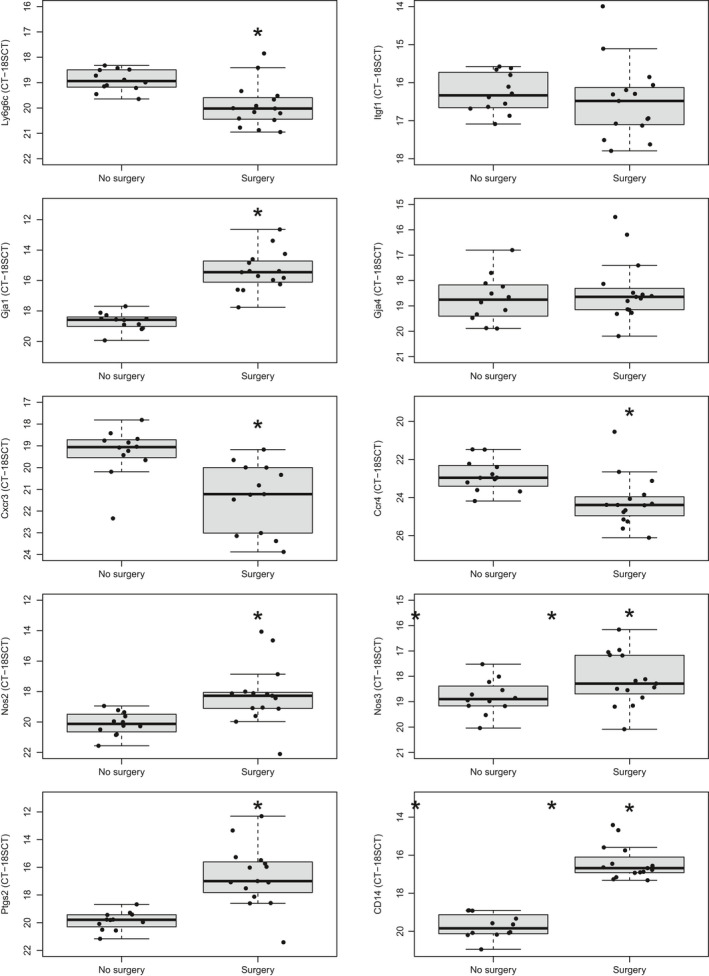
Change in gene expression by QRTPCR after surgery with prolonged anesthesia (no‐surgery, *n* = 12) and (surgery, *n* = 15). Data are expressed as CT–CT for 18S RNA. *Indicates *p* < 0.05 in comparison to no‐surgery group by the Student's *t* test

**FIGURE 4:**
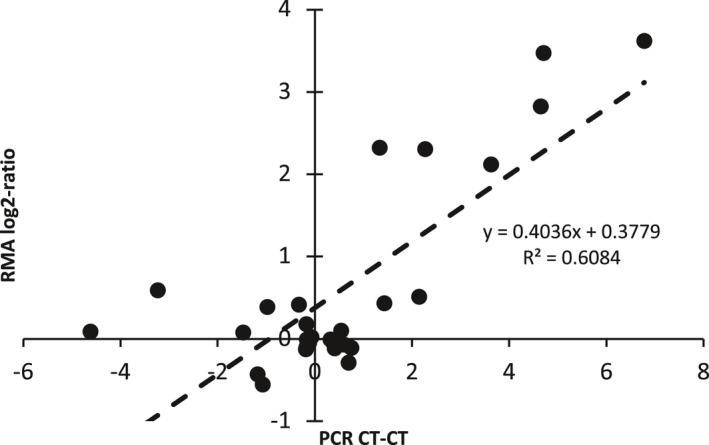
Correlation of group‐wise differential expression is analyzed using eBayes and corrected by the false discovery rate by PCR and microarray. Each point represents one gene that is investigated both in the microarray and by quantitative PCR. Pearson's correlation is reported

Selected genes indicative of activation of different immune cell lineages were confirmed using quantitative real‐time PCR (Figure 3). Notably, the expression of *Ccr4* (Th1) and *Cxcr3* (Th2) was reduced after surgery while the expression of the macrophage marker *Cd14* significantly increased. In addition, *Nos2* which is increased in activated macrophages was also higher in the surgery group. Other genes involved in inflammation (*Nos3* and *Ptgs2*) as well as *Gja1* were increased in the surgery group. Surgery had minimal effect on the expression of *Gja4* and *Itfg1*. This collectively indicates that macrophages may be activated, and that T cells and neutrophils are less likely to be involved. QRTPCR data correlated well to microarray data (Figure 4), which validates both the mRNA expressions found in our dataset and the hypothesis of immune cell activation.

### 3.7 Immunohistochemistry

**FIGURE 5:**
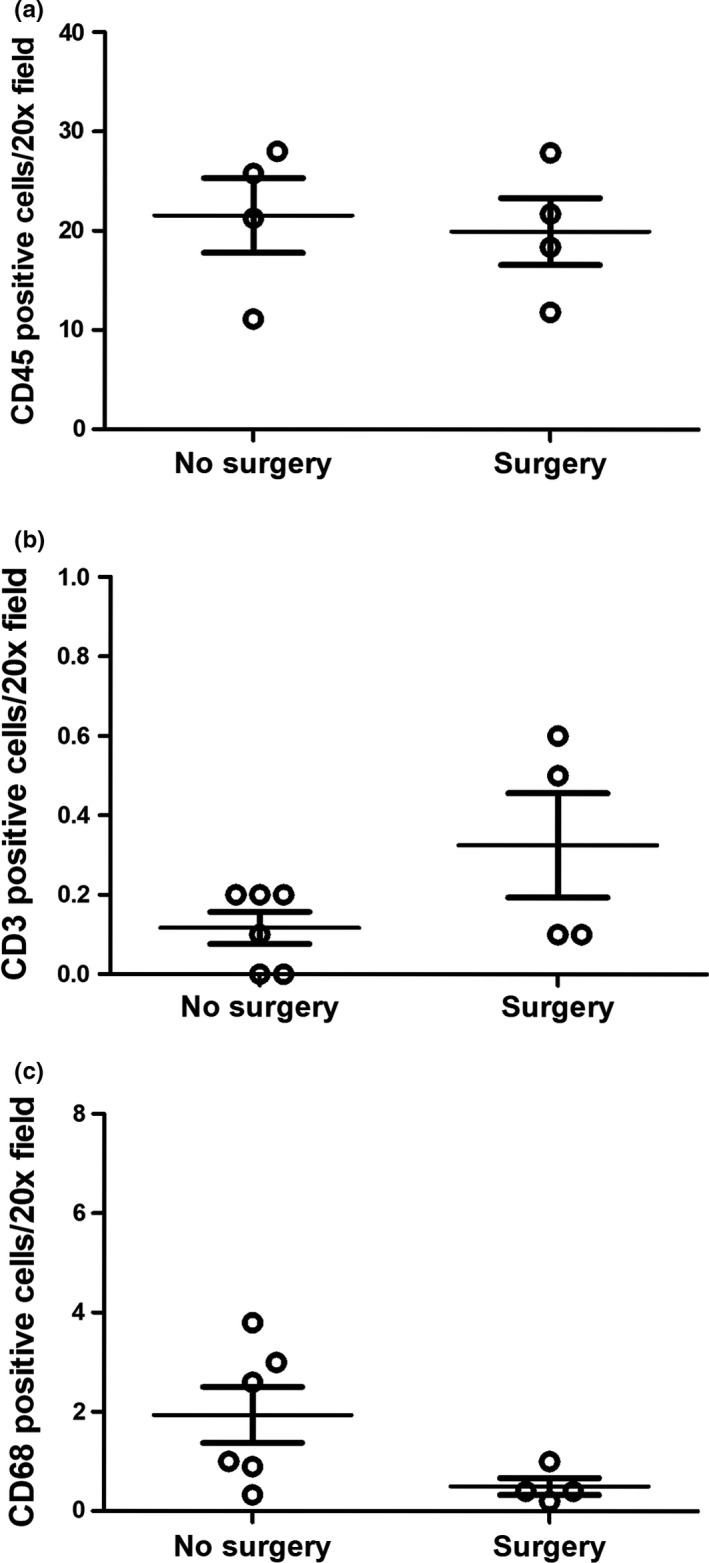
Quantification of renal immune cells by immunohistochemistry. A: CD45‐positive lymphocytes in rats that are killed quickly (*n* = 4), compared to rats that underwent surgery (*n* = 4). B: CD3‐positive T cells in rats that are killed quickly (*n* = 6) (*n* = 4). C: CD68‐positive macrophages in rats that are killed quickly (*n* = 6) compared to rats that underwent surgery (*n* = 4). Show no convincing accumulation of immune cells after long‐term surgery

Interestingly, CD45 immunohistochemistry did not show an increased number of total leukocytes in the kidneys. As expected from the microarray analysis, CD3‐positive T cells showed very low expressions but showed no difference between groups. Finally, CD68 did not identify an increased number of macrophages in the kidneys after prolonged surgery indicating that the increased expression of macrophage markers is related to activation of resident cells (Figure 5).

### 3.8 Sterile experiments

In the follow‐up experiments comparing fully sterile surgery with the aseptic technique which is standard procedure for acute experiments in rats we found that the marker genes for inflammatory cells were not different between the groups (Table 4). However, *Gja1* was lower in sterile conditions, and *Nos3* was higher, which may indicate a difference in endothelial activation.

**TABLE 4:** Sterile versus non‐sterile surgery

Gene	Ct–Ct Sterile	Ct–Ct Non‐sterile	*p* value
*Ccr4*	23.65 ± 0.42	23.44 ± 0.25	0.5698
*Cd14*	17.38 ± 0.32	16.53 ± 0.30	0.798
*Cxcr3*	20.60 ± 0.57	20.86 ± 0.57	0.404654
*Gja1*	17.21 ± 0.29	16.80 ± 0.58	0.0231*
*Gja4*	17.66 ± 0.46	16.95 ± 0.27	0.493
*Itfg1*	16.93 ± 0.26	16.92 ± 0.18	0.441
*Ly6g6c*	19.70 ± 0.44	20.49 ± 0.31	0.377994
*Nos2*	19.65 ± 0.38	19.54 ± 0.48	0.1906
*Nos3*	15.87 ± 0.34	16.92 ± 0.27	0.000705*
*Ptgs2*	17.11 ± 0.33	17.75 ± 0.66	0.798

Note

Differences in gene expression between rats that underwent prolonged anesthesia and surgery under sterile (*n* = 4) and non‐sterile (*n* = 4) conditions showing minor differences compared to the differential expressions seen comparing surgery to no‐surgery. Data were normalized to a housekeeping gene (18s RNA) and presented as Ct–Ct value. A smaller CT–CT value represents higher relative gene expression. All data are presented as mean ± SEM.

* Indicates *p* < 0.05 in comparison to sterile group by the Student's *t* test.

## 4 DISCUSSION

The major finding of the present study is that macrophages are likely activated in the kidney after prolonged surgery with anesthesia, but without direct renal insult or overt AKI. Both microarray and qPCR data showed substantial changes in renal gene expression after surgery. Significant difference was found between surgery and no‐surgery groups. No additional effect could be detected in insulin‐treated diabetic animals.

The higher level of CD14 in the surgery group suggested an increasing number of macrophages or monocytes, while the T‐cell markers Cxcr3 and Ccr4 were both reduced after surgery (Imai et al., 1999; Qin et al., 1998). Together, the data show that cell infiltration in kidney after surgery may be mainly macrophages and monocytes, while T helper cells are reduced or less activated. The apparent activation of monocytes is consistent with what was previously observed in acute kidney injury, but with actual kidney injury T‐cell populations are also increased (Doi et al., 2009). One study using two different AKI models showed that macrophages infiltrate into the kidney and that the phenotype of macrophages changes with time after surgical trauma. During the early stage, mainly M1 macrophages were found in the kidney which tend to be pro‐inflammatory and destructive. However, macrophages can also promote fibrosis and the scarring process in kidney by switching to anti‐inflammatory type M2 macrophages (Lv et al., 2017). Interestingly, distal surgical trauma has been found to induce macrophages infiltration across the blood–brain barrier in mice after stabilized tibial fracture surgery, suggesting a global role of macrophage infiltration in postoperative organ dysfunction (Terrando et al., 2011). This is in line with previous literature showing the important role of immune cells in AKI in various experimental models. The apparent reduction in T cells after surgery may also be a change that predisposes to further injury, since subpopulations of T cells are known to be protective and important for recovery after AKI (Gharaie Fathabad et al., 2020). Further studies are required to understand if this early infiltration of macrophages could predispose the kidneys to the development of postoperative AKI if subjected to a further insult.

In addition to the changes most probably connected to cell infiltration, surgical trauma‐induced changes in genes known to be involved in renal vascular function, such as *Gja1* and *Nos3*. The encoded protein of *Gja1* has an essential role in gap junction communication, but may also be involved in transferring antigens between antigen‐presenting cells and may thus be connected either to infiltration or early vascular changes (Mazzini et al., 2014; Paznekas et al., 2003). Interestingly, the expression of the gap junction protein *Gja4* was not changed by surgery in the original experiment, but the expression differed between sterile and nonsterile surgery, a finding that is hard to explain mechanistically and may be of interest for further investigation. *Nos3* or eNos is expressed in endothelial cells and regulates vascular tone, and was increased in the surgery group, indicating early changes that could cause dysregulation of vascular function (Bauer & Sotníková, 2010).

The results demonstrate that surgical trauma and prolonged anesthesia with mechanical ventilation have pronounced effects on renal gene expression, which need to be kept in mind when measuring the change in renal gene expression after any invasive procedure. Furthermore, the microarray data showed that a large number of biological processes are implicated in the genetic response to surgical trauma and that these are at least in part similar to the genetic effects of AKI (Hultstrom et al., 2018). Therefore, in future physiological studies we need to specify if the observed effect is from experimental procedure or surgical trauma itself.

It is important to note that this model does not fulfill the clinical AKI criteria. Renal blood flow, was investigated in the original publication (Mitrou et al., 2015), and urine production and glomerular filtration rate have been published previously (Lau et al., 2009). However, the comparison to previously known AKI genes showed increased expression of lipocalin‐2 (LCN2), in the AKI field often referred to as neutrophil gelatinase‐associated lipocalin (NGAL), which may indicate early renal tissue injury (Mishra et al., 2003) or an epithelial stress response (Fahling et al., 2019). It should be noted that LCN2 can also be released from activated neutrophils, although other markers of neutrophil activation were not prominently upregulated, and inflammatory cell numbers were not increased in total.

To evaluate the effect of surgery, the resident immune cell in the kidney under normal physiological conditions needs to be identified. In healthy kidney, the resident immune cells are mainly dendritic cells (DCs) and macrophages, together they compose the renal mononuclear phagocytic system, which plays a central role in kidney homeostasis (Nelson et al., 2012). Both cell types have high expression of pattern recognition receptors and are involved in innate and adaptive immune response in defense against infection. During surgery, macrophages appear to be rapidly activated or recruited by pro‐inflammatory chemokines. Therefore, in situ imaging studies with long preparatory invasive procedures could be particularly prone to overestimating the normal renal immune cell population.

A second question that we address in the present study is the effect of bacterial contamination during surgery on renal gene expression and immune cell infiltration. In the clinical setting, sepsis is the main cause of AKI among intensive care patients (Uchino et al., 2005). Bacterial contamination could be introduced by non‐sterile procedures (Chelemer et al., 2002; Triulzi et al., 1992). There were few differences between the sterile and non‐sterile groups that were performed in parallel that may indicate some effect on endothelial cells. However, the sterile and non‐sterile groups were generally similar to the surgery group of the microarray study indicating that it is indeed prolonged anesthesia and surgical trauma that induce the gene expression changes, and not bacterial contamination. STZ diabetes has been shown to induce gene expression changes in various models and the reason they are not significant in the present study may be related to differences in the diabetic models with regard to insulin treatment and hyperglycemia as well as the relatively modest changes compared to the effect of surgery and anesthesia. Finally, anesthesia may be a direct risk factor for kidney injury. Anesthesia is known to reduce cardiac output and total peripheral resistance, as well as renal blood flow (Papper & Ngai, 1956). However, these effects cannot be separated from the surgical trauma in the current study.

In conclusion, surgical trauma and long‐term anesthesia without direct kidney injury are associated with marked changes in renal gene expression likely connected to monocyte or macrophage activation. This may make the kidneys more prone to develop AKI upon further insult and may provide some explanation for the increased risk for AKI in patients undergoing surgery.

## CONFLICT OF INTEREST

The authors declare no conflict of interest.

## AUTHOR CONTRIBUTIONS

NM and MH conceived the study. All authors participated in design and planning. MH, DP, MBA, CGC, and NM performed the experiments. All authors participated in data analysis and interpretation. MH and DP drafted the manuscript. All authors edited the manuscript for intellectual content and approved the final version.

## Data Availability

The microarray dataset is available at Array Express with accession number: E‐MTAB‐3661 (http://www.ebi.ac.uk/arrayexpress/). All other data are presented in the manuscript and available from the authors upon reasonable request.
